# Supplementary figures and images for: Methanogenesis marker 16 metalloprotein is the primary coenzyme M synthase in Methanosarcina acetivorans
Source: PLoS Genet. 2025 May 2;21(5):e1011695. doi: 10.1371/journal.pgen.1011695 (PMC12068725; doi:10.1371/journal.pgen.1011695)

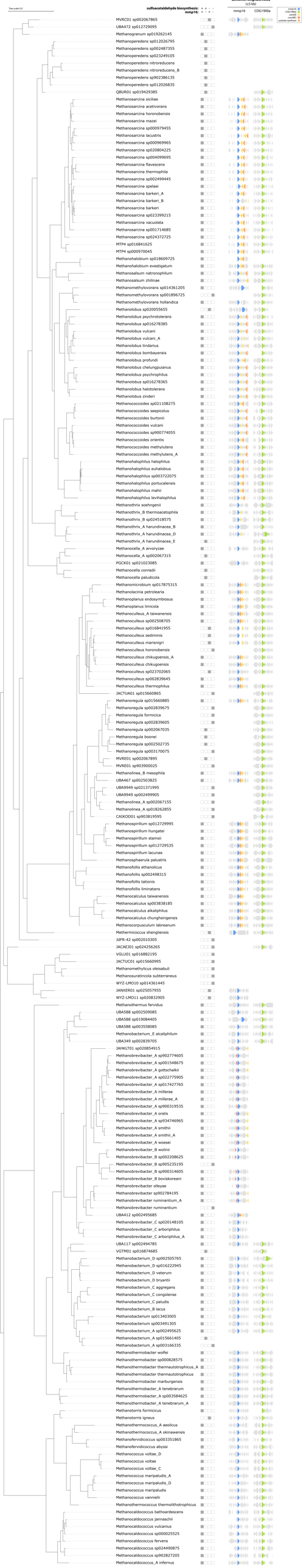

Supplement: S1 Fig — (PDF) [file pgen.1011695.s002.pdf]
