# Supplementary material for: Methanogenesis marker 16 metalloprotein is the primary coenzyme M synthase in Methanosarcina acetivorans
Source: PLoS Genet. 2025 May 2;21(5):e1011695. doi: 10.1371/journal.pgen.1011695 (PMC12068725; doi:10.1371/journal.pgen.1011695)

**Supplementary Table S2:** Predicted mutations of genome re-sequencing analyzed by Breseq. The expected deletions were observed in ∆*cs* (MA3297/MA_RS17200), ∆*comDE* (MA3298/MA_RS17205), ∆MMP16 (MA3299/MA_RS17210), and ∆*L-ASST* (MA1821-22/MA_RS09480-85). An additional G->T point mutation was observed in MA_RS02405 in the ∆*comDE* strain. The impact of this additional point mutation was not evaluated.

**∆*cs* (MA3297/MA_RS17200)**
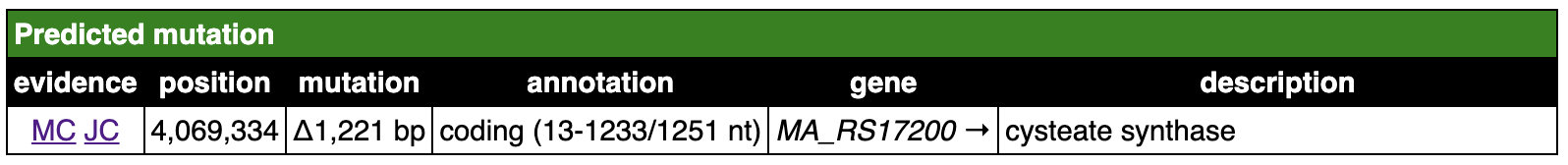


**∆*comDE* (MA3298/MA_RS17205)**


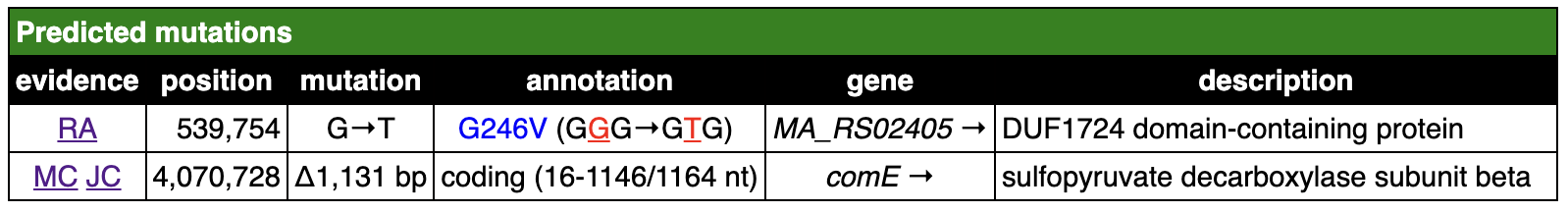


**∆MMP16 (MA3299/MA_RS17210)**
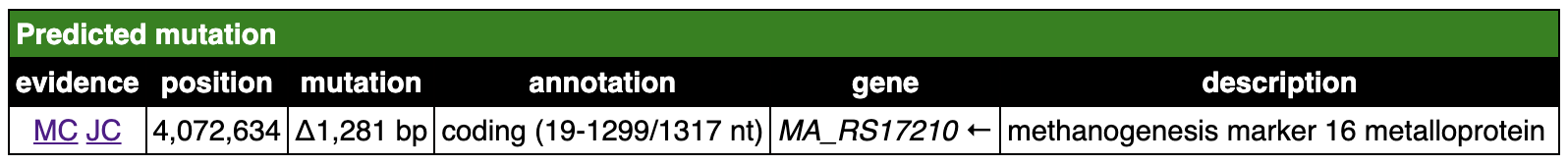


**∆*L-ASST* (MA1821-22/MA_RS09480-85)**
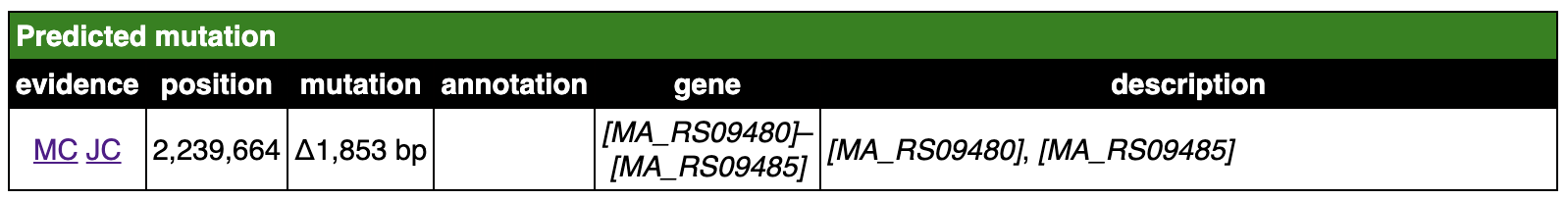

Supplement: S2 Table — The expected deletions were observed in ∆ cs (MA3297/MA_RS17200), ∆ comDE (MA3298/MA_RS17205), ∆ MMP16 (MA3299/MA_RS17210), and ∆ L-ASST (MA1821–22/MA_RS09480–85). An additional G- > T point mutation was observed in MA_RS02405 in the ∆ comDE strain. The impact of this additional point mutation was not evaluated. (DOCX) [file pgen.1011695.s003.docx]
