# Supplementary material for: Methanogenesis marker 16 metalloprotein is the primary coenzyme M synthase in Methanosarcina acetivorans
Source: PLoS Genet. 2025 May 2;21(5):e1011695. doi: 10.1371/journal.pgen.1011695 (PMC12068725; doi:10.1371/journal.pgen.1011695)
Supplement: S3 Table — Total number differentially expressed in black, total number up-regulated (in the row label relative to the column label) shown in blue, total number down-regulated shown in red. All DESeq2 data presented in Supplementary S4 Table. (DOCX) [file pgen.1011695.s005.docx]

**Supplementary Table S3:** Protein-coding genes found to be differentially expressed by DESeq2 analysis, using a q-value cutoff of < 0.001 and an absolute value of log2(fold change) > 1. Total number differentially expressed in black, total number up-regulated (in the row label relative to the column label) shown in blue, total number down-regulated shown in red. All DESeq2 data presented in **Supplementary Table S4**.

**
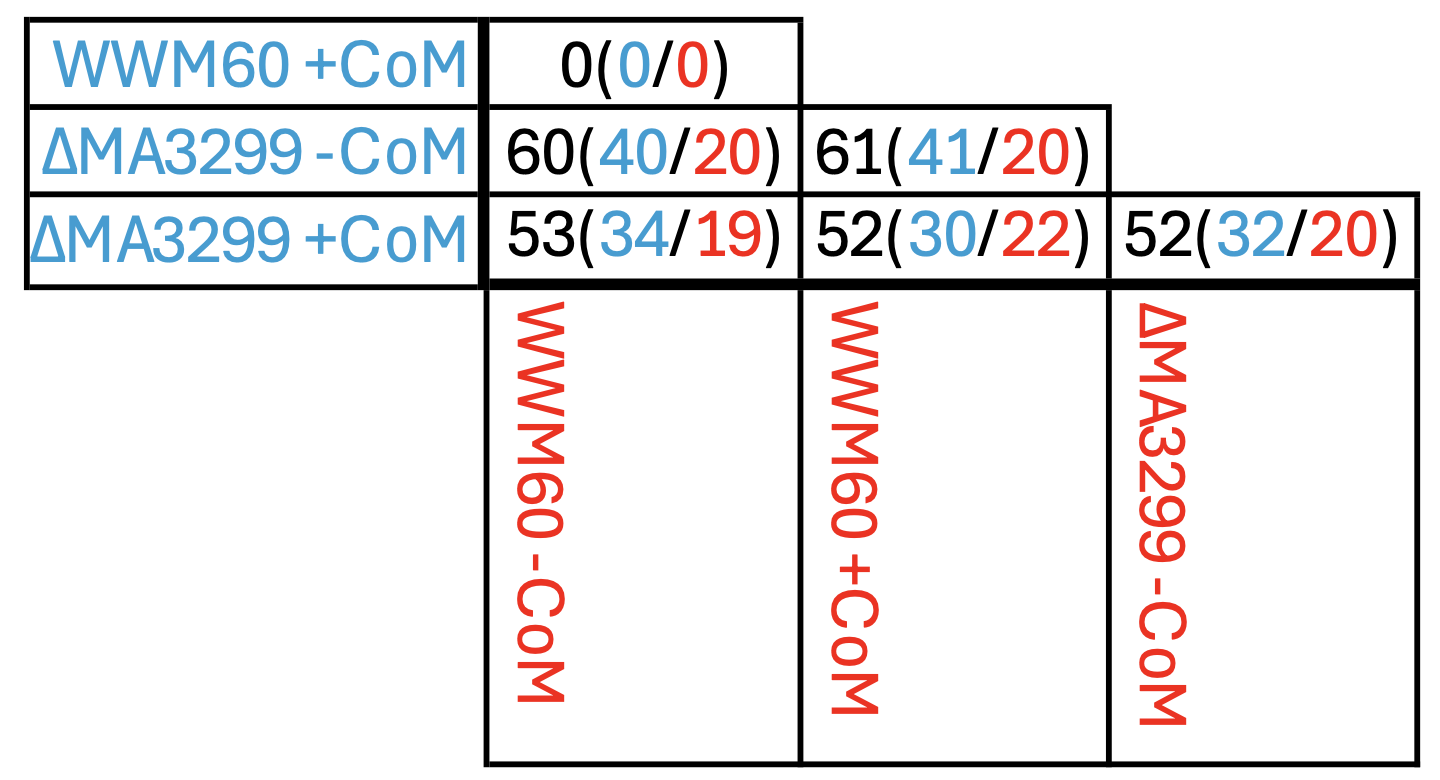
**
