# Supplementary material for: Methanogenesis marker 16 metalloprotein is the primary coenzyme M synthase in Methanosarcina acetivorans
Source: PLoS Genet. 2025 May 2;21(5):e1011695. doi: 10.1371/journal.pgen.1011695 (PMC12068725; doi:10.1371/journal.pgen.1011695)
Supplement: Attachment — Submitted filename: Response to Reviewers.docx [file pgen.1011695.s017.docx]

Please find our responses to the editor’s and reviewer’s comments in green below. All line numbers correspond to the marked-up copy manuscript with the changes highlighted.

**Additional Editor Comments:**

The reviewers found the study very valuable and have included minor corrections to be made in the manuscript. However, it will be critical to describe the sequencing methods more clearly and provide the SRA data for this study.

Thank you for positive feedback. We have described the sequencing methods in detail in the methods section of the revised manuscript (see lines 445-460) and also added the SRA entries as a BioProject number in the data availability section.

**Reviewers' comments:**

Reviewer's Responses to Questions

**Comments to the Authors:
Please note here if the review is uploaded as an attachment.**

Reviewer #1: Interesting contribution to our understanding of coenzyme M biosynthesis in methanogens. The reviewer has only two comments:

Figure 2.B Please check for correctness. e.g. Methanothermobacter marburgensis is not a member of the Methanococcales.

Thank you for this correction!  We have modified Fig 2B to place *M. marburgensis* into the *Methanobacteriales* where it should be, and changed the figure legend to reflect this change.

Why ComF/MMP16 rather than MMP16 in Fig. 2A". It would be helpful if in the text to Fig. 2 information with respect to the distribution of the L-aspartate semialdehyde sulfotransferase would also be given. Do all listed archaea contain the corresponding gene(s)?

We had included ComF in the label ComF/MMP16 to match the other labels in this figure, such as ComDE/Sulfopyruvate decarboxylase. To avoid confusion, we have changed the labels in Fig 2A to only be MMP16.

With regard to the presence of L-ASST in archaea, we have added a line to the figure 2 legend the text: “Note: L-ASST is not present in many methanogens, and is also found in many non-MCR containing archaea (**Table S1** and **Fig. S1**), supporting the notion that its primary role is not connected with methanogenesis.”

Authors Summary: Omit last sentence: "Since MMP16 is widely distributed in, and unique to, methanogens it is ideal candidate for the design of anti-methanogen chemical inhibitors". This sentence is misleading for two reasons: (i) with respect to "unique to" MMP16 is also present in methanotrophic archaea and absent in some Methanobrevibacter species; With respect to "ideal candidate", the authors have shown that inhibition (deletion) of MMP16 only leads to a CoM auxotroph in the absence of sulfide, which in anaerobic environments is abundant.

These are indeed good objections that the reviewer brings up, so we have removed that sentence.

Reviewer #2: The manuscript from Chadwick et al., resolves a long-standing conflict in the methanogenesis field regarding the biosynthetic origin of the essential cofactor coenzyme M (CoM). In particular, prior studies suggest a functional role of the gene production ComF as the enzyme that catalyzed the conversion of sulfoacetaldehyde to CoM in the last step in type 1 methanogens but genetic deletions of the corresponding gene were still viable. In this more detailed studied, the authors show that the comF deletion variant do have a growth phenotype, especially in the absence of exogenous sulfide, and that a housekeeping sulfurtransferase could compensate for the loss of comF, thereby resolving the previously conflicting reports.

The work is nicely carried out and well within the scope of PloS Genetics. My only request is that the authors include the Alphafold2 models that are alluded to in Lines 325-326 as Supplemental Figures as they are important to support their theory that the housekeeping sulfurtransferase may have a distinct mechanism from ComF.

We have referenced the Alphafold2 model presented in Fig S5 in the text (line 334) as requested by the reviewer.

Reviewer #3: The study by Chadwick et al investigates the involvement of the protein MMP16 in the biosynthesis of Coenzyme M, which is an essential cofactor for all methanogenic archaea. While there was prior evidence that MMP16 is involved in this step, there were also contradictory experimental studies that suggested a second, rescue pathway, for the catalysis of this reaction. This present manuscript expands on prior work by showing that MMP16 is a bona fide CoM synthase and elucidates some of the possible rescue pathways.

The authors are commended for the biochemical sleuthing. To me, they could convincingly show that the MMP16 protein is the bona fide CoM synthase, and that high sulfide concentrations in combination with the activity of L-ASST can rescue CoM synthesis. This is rounding up several previous reports in the field with seemingly contradictory results and closes a chapter that has not been concludingly answered in the past. I think this is a beautiful piece of work and I congratulate the authors for their creativity in experimental design and aptitude in conducting them.

I have three main concerns:
L350-380: the discussion is rather short and does not tie together the results obtained by others and the results in this paper; the strength of this paper is that it removes contradictions and is able to explain some of the previously obtained results that seemed unlogical. These are mentioned during the introduction and results but for the sake of clarity I recommend expanding the discussion to include those here.

We thank the reviewer for this comment. We were debating whether to discuss previous work in the field that our study directly, contradicts. Ultimately, we decided against it as it we did not want to cast aspersions on any prior studies and our work conclusively demonstrates the role of MMP16 as a coenzyme M synthase, it and of itself. That said, to address reviewer’s comment, we have added the following to the discussion section: ”Regardless of the exact nature of the back-up CoM biosynthesis pathway, our results clearly demonstrate that MMP16 is primary CoM biosynthesis enzyme *in vivo*, and explain the conflicting results in the literature.”

L382 and following: I find the entire Materials part pretty sparse, a lot “as described previously”. While I understand that it saves time and space for the writer it can be difficult for (inexperienced) readers to find back all these procedures that were described elsewhere. I urge the authors to reconsider this way of describing procedures and rather include all recipes, procedures etc. in the primary text, or in a supplementary material paragraph.

We thank the reviewer for pointing this out. Our intention was not to obfuscate any methodological details but to be concise. We have taken their comment to heart and the specific areas where the Materials section was lacking is enumerated below in the “Minor comments” area, and we have addressed each of these issues as they arise there.

L439, 453: Primary research data are not made available to the reviewer. I think this is not ok. Please let the reviewer see the SRA entry during reviewing.

We apologize that the reviewer was not able to access the raw fastq files during their review, but they have now been made public as part of the BioProject: PRJNA1245437 which now appears in the data availability statement.

Minor comments:
L49: it is an ideal candidate

This sentence has been removed per reviewer #1’s request

L330: I think the reader could benefit from a display item for this in the main text, e.g. Suppl Fig S6

We agree this is a helpful visual, and an initial draft of the manuscript did have this display item in the main text. However, we ultimately decided against it since we already had 7 main text figures. Hopefully, since this is mainly a digital publication, the identification of Supp Fig S6 be the interested reader is not too onerous.

L374: did the authors try to grow the deltaL-ASST double mutant in the presence of methionine (and CoM)?

Yes, methionine is not expected to be essential for growth in the ∆L-ASST double mutant as a prior study (<https://onlinelibrary.wiley.com/doi/full/10.1111/mmi.12832>) has demonstrated that there is an entirely different pathway for methionine biosynthesis in *Methanosarcina.* To address this comment, we have added text to this effect on line 284 when this data was first presented.

L399: with using, remove one

Thank you, we have fixed this line in the text.

L405: please list HS medium recipe here. I know it was described elsewhere but I think it’s good practice to include as many key procedures as possible.

We have now added the recipe for HS media in full from lines 408-416

L410: colonies were screened by PCR? Which primers?

We have added a reference here to Supplemental Table S6 on line 422, which has a list of all the primers and how they were used for this work.

L433: what is a saturated culture? A stationary culture?

We have changed saturated culture to stationary phase culture as that is a more common phrase.

L436: there is by far too little information on the sequencing. Which sequencing kit, which platform (Myseq, Hiseq, etc), how many reads per sample, how were samples quality controlled, how were adapters removed etc. Please expand to fully explain the method.

We have added the DNAse, Illumina kits, instrument model, and the bioinformatics tool used for demultiplexing, QC and adapter removal  to the methods of the sequencing for both DNA and RNA sequencing (lines 445-460).

L448: how was the transcriptome collected? Please describe details of the technology and chemistry. Please list statistics of runs. How many reads per sample etc.

The information for the technology/chemistry was added to the methods as described above.  We have added an additional Supplemental Table S8 that outline the statistics of the transcriptome experiment.

**Have all data underlying the figures and results presented in the manuscript been provided?**
Large-scale datasets should be made available via a public repository as described in the *PLOS Genetics* [data availability policy](http://track.editorialmanager.com/CL0/http:%2F%2Fjournals.plos.org%2Fplosgenetics%2Fs%2Fdata-availability/1/010f0195f7128690-844c2b94-d11c-45ec-a255-721cd21d0d60-000000/6zBUW2gkbGeAwrQgkKXPvCO9urnO-A3DppeAMnCOwsU=204), and numerical data that underlies graphs or summary statistics should be provided in spreadsheet form as supporting information.

Reviewer #1: Yes

Reviewer #2: Yes

Reviewer #3: **No:** The SRA entries have not been provided.

We have added the SRA entries as Reviewer #3 requested (BioProject number in the data availability section).
